# Effects of Hepatitis C Virus Elimination by Direct-Acting Antiviral Agents on the Occurrence of Oral Lichen Planus and Periodontal Pathogen Load: A Preliminary Report

**DOI:** 10.1155/2021/8925879

**Published:** 2021-11-11

**Authors:** Yumiko Nagao, Masahide Tsuji

**Affiliations:** ^1^Department of Public Health, Graduate School of Medicine, Juntendo University, Tokyo 113-8421, Japan; ^2^Liver Center, Saga University Hospital, Nabeshima, Saga 849-8501, Japan; ^3^Tsuji Dental & Oral Surgery Clinic, Shiragane-machi, Omuta, Fukuoka 836-0052, Japan

## Abstract

**Objective:**

The association between hepatitis C virus (HCV) and oral lichen planus (OLP) is well known, but the association with periodontal disease has been reported less often. The purpose of this study was to investigate the effects of periodontal bacteria and OLP lesions before and after HCV elimination. *Subjects and Methods*. The subjects were four OLP patients (mean age 72.5 years) with HCV infection. Six types of periodontal bacteria (*Aggregatibacter actinomycetemcomitans, Prevotella intermedia*, *Porphyromonas gingivalis*, *Tannerella forsythia*, *Treponema denticola*, and *Fusobacterium nucleatum*) were quantified in saliva, and changes in OLP were examined before and after elimination of HCV by antiviral therapy. Biochemical blood tests also were performed.

**Results:**

The total number of periodontal bacteria, the numbers of *P. gingivalis*, *T. forsythia*, *T. denticola*, and *F. nucleatum*, and the risk of presenting with the red-complex bacteria (*P. gingivalis*, *T. forsythia,* and *T.denticola*), leading to periodontal disease progression, decreased after HCV elimination. OLP disappeared in three of the four patients and decreased in the other after sustained virological responses (SVRs).

**Conclusion:**

HCV elimination not only improved OLP lesions but also reduced the number of periodontal pathogens and the amount of red-complex periodontal pathogens.

## 1. Introduction

Hepatitis C virus (HCV) infection affects approximately 71 million people worldwide and is a leading cause of chronic liver disease and liver cancer deaths [1, 2]. In Japan, liver cancer accounts for about 30,000 deaths per year, 80% of which are caused by hepatitis B virus (HBV) and HCV [3]. HCV-infected patients in Japan are generally older than in other countries and are characterized by a high proportion of patients with complications such as cirrhosis and liver cancer [4, 5]. Since 2014, the treatment of chronic hepatitis C has been revolutionized by the advent of oral direct-acting antiviral agents (DAAs) [6]. Because DAA treatment has enabled the elimination of HCV, in May 2016, the World Health Organization (WHO) aimed to eliminate viral hepatitis as a major public health threat by 2030 [7, 8].

HCV infection is known to cause manifestations other than liver disease [9]. Oral lichen planus (OLP), which is recognized as an oral potentially malignant disorder (OPMD), is one of these extrahepatic manifestations [10, 11]. In addition to glucocorticoids and immunosuppressive drugs (cyclosporine), the efficacy of tacrolimus, clobetasol [12], pimecrolimus [13], and glycyrrhizin [14] has been reported for the treatment of OLP. We have reported that OLP can be cured when HCV is eliminated by antiviral treatments, such as with interferon (IFN) [15] and DAA [16–19]. Achieving a sustained virological response (SVR) has been shown to reduce not only liver cancer deaths and liver disease-related deaths but also extrahepatic mortality [20, 21].

Periodontal disease is associated with systemic health [22] and with liver disease, particularly nonalcoholic steatohepatitis (NASH) [23]. As a new biomarker involved in periodontal inflammation, galectin-3 has recently been shown to have the potential to discriminate between periodontitis and periodontal health [24]. We reported previously that there is an association between the severity of periodontal disease and the progression of viral liver disease [25] and that the so-called red-complex bacterial species (*Porphyromonas gingivalis*, *Tannerella forsythia*, and *Treponema denticola*) which are associated with severe periodontal disease are also associated with liver cirrhosis [26]. However, the changes in periodontal pathogens with and without HCV elimination have not been reported. Here, we report, as a preliminary study, the changes in periodontal species after HCV elimination by antiviral therapy in four OLP patients. The aim of this study was to investigate the effect of periodontal bacteria and OLP lesions before and after HCV elimination.

## 2. Subjects and Methods

### 2.1. Patients

The subjects were those who met the following eligibility criteria: (i) diagnosed with chronic hepatitis C and oral lichen planus; (ii) achieved SVR by DAA treatment; (iii) underwent saliva collection and oral mucosal examination at least twice before and after DAA treatment; (iv) positive for HCV antibody and HCV RNA, but negative for hepatitis B surface antigen (HBsAg); (v) any gender; and (vi) obtained written consent for this study. Exclusion criteria are as follows: (i) subjects who had not given consent; (ii) subjects who had not received DAA treatment; (iii) subjects who had not achieved SVR with DAA treatment; (iv) complications of other liver diseases (chronic hepatitis B, autoimmune hepatitis, primary biliary cirrhosis, primary sclerosing cholangitis, Wilson's disease, etc.); and (v) the presence of hepatocellular carcinoma (HCC) (except for patients with no recurrence for more than one year after treatment for HCC).

The subjects were four OLP patients (two males and two females) with HCV infection who consented to oral examinations and saliva collection before DAA treatment and after SVR. The ages of the subjects ranged from 65 to 77 years, with a mean of 72.5 ± 5.4 years. The background information of the subjects is shown in Table 1. The four patients visited Saga University, Kurume University, or Tsuji Dental and Oral Surgery Clinic between May 2013 and June 2015. The oral and liver diseases of each patient were diagnosed by an oral surgeon and a hepatologist. Three of the four patients received DAA treatment with daclatasvir (DCV; NS5A inhibitor) and asunaprevir (ASV; NS3 protease inhibitor), and the other received DAA treatment with sofosbuvir (SOF; NS5B inhibitor) and ribavirin (RBV), all of which resulted in SVR.

### 2.2. Examination of Oral Mucosal Disease

Before receiving DAA treatment and 24 weeks after SVR (SVR24), the oral mucosae were examined using a headlamp (Welch Allyn Ltd.). The diagnosis of OLP was made clinically and/or histopathologically. Information regarding the daily frequency of tooth brushing, smoking habits, and alcohol consumption was collected from the patients.

### 2.3. Saliva Sample Collection

A total of 1 mL of saliva was collected from the patients following chewing a nonflavored gum for 5 min. All samples were immediately sent to the Health Examination Laboratory (BML, Inc., Tokyo, Japan) for bacterial testing [27]. Saliva was collected twice from each patient, before receiving DAA treatment and after SVR24. The subjects did not change their lifestyle before or after DAA treatment, did not receive active treatment for periodontal disease, and did not receive antibiotic treatment.

### 2.4. Identification of Periodontitis Bacteria and Detection of *P. gingivalis* fimA Genotypes

The total number of bacteria in saliva and the counts of six types of periodontal pathogens (*Aggregatibacter actinomycetemcomitans; Prevotella intermedia; P. gingivalis; T. forsythia; T. denticola;* and *Fusobacterium nucleatum*) were quantified using the modified polymerase chain reaction (PCR) Invader assay, as described previously [28]. The ratio of the number of periodontal disease bacteria to the total number of bacteria was examined (e.g., salivary *P. gingivalis* ratio (ratio: *P. gingivalis* counts/total bacteria counts)). Then, those with a high percentage of periodontal disease bacteria were judged to be at risk [26]. Based on the ratio of each periodontal pathogen to the total bacterial count, the following criteria were used to assess the risk: (i) *A. actinomycetemcomitans*; no risk: < 0.005% and risk: ≥ 0.005%; (ii) *P. intermedia*; no risk: < 0.09% and risk: ≥ 0.09%; (iii) *P. gingivalis*; no risk: < 0.09% and risk: ≥ 0.09%; (iv) *T. forsythia*; no risk: < 0.09% and risk: ≥ 0.09%; (v) *T. denticola*; no risk: < 0.09% and risk: ≥ 0.09%; and (vi) *F. nucleatum*; no risk: <5% and risk: ≥ 5%. The red-complex bacteria were divided into two categories based on the aforementioned risk criteria: cases in which the percentage of at least one of the three red-complex bacteria increased (with risk) and cases in which there was no risk at all (without risk). *P. gingivalis-*specific fimA genotypes, ranging from types I to V, were detected in all patients. The periodontitis bacteria were identified in each patient, before receiving DAA treatment and after SVR24.

### 2.5. Serological Assays

All subjects were assessed for white blood cell (WBC) counts, red blood cell (RBC) counts, hemoglobin (Hb), platelet (Plt) counts, aspartate aminotransferase (AST), alanine aminotransferase (ALT), lactate dehydrogenase (LDH), alkaline phosphatase (ALP), total protein (T. pro), albumin (Alb), total bilirubin (T. Bil), fasting blood glucose, HbA1c levels, total cholesterol (T. cho), and alpha-fetoprotein (AFP). All the biochemical parameters were measured by standard clinical methods using venous blood samples taken the morning after a 12 h overnight fast.

### 2.6. Evaluation of Liver Diseases

Anti-HCV was measured using a chemiluminescent enzyme immunoassay kit (Lumipulse II HCV; Fujirebio). HCV RNA in serum was analyzed using a standardized automated quantitative PCR (COBAS AMPLICOR HCV MONITOR v2.0 Test; COBAS^®^ AmpliPrep/COBAS^®^ Taq-Man^®^ HCV Test; Roche Molecular Diagnostics) as reported previously [29]. Similarly, HCV genotypes were determined by PCR, as reported previously [30]. Ultrasonography was performed on all patients to examine the shape of the liver and to identify the lesions in the organ. Computed tomography was performed on all patients.

### 2.7. Ethics Approval

The study was conducted according to the guidelines of the Declaration of Helsinki and approved by the Ethical Committee of Saga University (reference no. 2015-02-16 and 29–71) and the Ethics Committee of Kurume University (reference no. 12240). Written informed consent for participation in the study was obtained from each patient.

## 3. Results

### 3.1. Periodontitis Bacteria and Detection of *P. gingivalis* fimA Genotypes

Table 2 shows the changes in periodontal disease bacteria from before DAA treatment to after SVR. After SVR, the total number of periodontal bacteria and the numbers of *P. gingivalis*, *T. forsythia*, *T. denticola*, and *F. nucleatum* decreased. After SVR, the number of *A. actinomycetemcomitans* remained unchanged and the number of *P. intermedia* increased. Two patients (50%) were at risk for at least one of the three types of red-complex bacteria before DAA treatment, but none (0%) after SVR (Table 2). The type of *P. gingivalis* fimA did not change with DAA treatment: one patient had type II, one had type III, and the remaining two were undetectable.

### 3.2. OLP

Four patients did not receive any specific drug therapy for OLP during DAA treatment but, after SVR24 with DAA treatment, OLP lesions disappeared in three of the four OLP patients and improved in the other (Table 1).

### 3.3. Liver Diseases

All subjects achieved SVR at the end of treatment. A comparison of the biochemical data at baseline and SVR24 is shown in Table 2. At SVR24, the serum AST, ALT, LDH, ALP, and AFP levels decreased to normal and the Alb levels increased. The BMI did not change before and after treatment. No clinical adverse events were observed in the patients.

## 4. Discussion

DAA treatment of hepatitis C is a remarkable development. The elimination of HCV by DAA treatment has long-term benefits [31] and leads to improved outcomes at all stages of liver disease [32]. HCV elimination with DAA treatment shows the following benefits: reduction of liver fibrosis and portal hypertension [33], reduced incidence of HCC [34], reduced liver-related mortality [35], reduced incidence of diabetes [36], reduced incidence of cardiovascular disease [37], reduced risk of developing mixed cryoglobulinemia, glomerulonephritis, and lichen planus [38], improved patient quality of life [39], and reduced all-cause mortality [35].

In this study, we investigated the effects of DAA on periodontal disease and OLP. Not only did OLP lesions disappear or improve when HCV was eliminated but also the number of periodontal bacteria and the content of red-complex periodontal bacteria decreased. This is the first report of a decrease in the number of periodontal disease bacteria following HCV elimination. The conventional IFN treatment of OLP patients with HCV infection has the disadvantage that OLP often worsens during treatment and the treatment cannot be completed [40–42]. However, DAA therapy has fewer side effects than IFN therapy and results in an SVR rate of over 90% [43–45]. We reported previously that DAA treatment of HCV-infected OLP does not worsen OLP lesions during treatment and that OLP improves or is cured after HCV elimination [16, 17, 19]. Su et al. reported that elimination of HCV also reduced the incidence of oral cancer; in a nationwide population study, HCV-infected individuals had a significantly increased risk of both OLP and oral cancer compared to noninfected individuals and antiviral therapy with pegylated IFN (PegIFN) and RBV significantly reduced the risk of HCV-related oral cancer [46]. We reported previously a high prevalence of HCV infection in oral cancer [47] and multiple primary squamous cell carcinoma of the head and neck [48, 49]. HCV-positive OLP lesions are considered to be at high risk of malignant transformation [50]. A genome-wide association analysis (GWAS) analysis of Japanese HCV-infected patients showed that HLA-DR/DQ genes were significantly associated with the development of HCV-related OLP [51], which supported previous reports [52]. Furthermore, we identified rs884000 of neuropilin-2 (*NRP2*) and rs538399 of insulin-like growth binding protein factor 4 (*IGFBP4*) as novel associations, indicating that these two SNPs may be involved in the malignant transformation of HCV-infected OLP [51]. Further studies are needed to clarify the link between HCV and oral carcinogenesis, but there is no doubt that HCV elimination is beneficial.

HCV infection is known to stimulate the immune system, causing cytokine production and chronic inflammation [53]. HCV is involved in vascular inflammation through the activation of tumor necrosis factor alpha (TNF*α*), causing the activation and adhesion of inflammatory cells in the blood vessels [54]. HCV core proteins cause immune activation, inflammation, and tumorigenesis through the signal transducer and activator of the transcription 3 (STAT 3) pathway [55]. On the other hand, lipopolysaccharide (LPS), a component of the cell wall of periodontopathogenic bacteria, invades gingival tissues, activates neutrophils and macrophages, and produces various proinflammatory factors such as interleukin- (IL-) 1*β*, IL-6, TNF*α*, and matrix metalloproteinases (MMPs) [56]. Gingipain, a cysteine protease produced by *P. gingivalis*, a major periodontopathogenic bacterium, activates MMP precursors [57], and *P. gingivalis* fimbriae act on macrophages and gingival fibroblasts to induce the production of inflammatory cytokines [58]. Azatyan et al. reported that oral fluid cytokine levels were significantly higher in patients with HCV, HBV, and HIV than in controls [59].

HCV infection is associated with a higher incidence of dental caries and periodontal disease than in healthy individuals, a trend that is likely to become more pronounced as liver fibrosis progresses. Coates et al. pointed out a trend of poor periodontal disease in hepatitis C patients, although this was not statistically significant [60]. We reported previously that the following five factors were associated with periodontal disease in 351 patients with liver disease attributable to HBV or HCV: Plt count <80,000, daily tooth brushing, current IFN treatment, age >65 years, and obesity [25]. The adjusted odds ratios for these factors were 5.80, 3.46, 2.87, 2.50, and 2.33, respectively, and were statistically significant. The periodontal pathogens' composition is generally influenced by aging [61]. Toljić et al. reported that HIV infection significantly increases the bacterial load in the oral cavity and alters the pattern of microflora composition during the aging process [61]. We also reported that the prevalence of fimA genotype II was higher in cirrhotic patients with advanced fibrosis than in chronic hepatitis [25] and that the red complex was associated with cirrhotic patients [26].

The reasons for the decreased risk of the red complex after HCV elimination in this study may be the reduction of inflammatory cytokines in the oral cavity, improvement of insulin resistance, improvement of OLP lesions, and involvement of the gut microbiota. Recently, it has been reported that disruption of the gut microbiota (so-called dysbiosis) is associated with liver diseases, such as nonalcoholic fatty liver (NAFL) and NASH [62], liver cirrhosis [63], primary biliary cholangitis [64], and HCV-related liver disease [65–67]. It has been reported that gut microbiota dysbiosis in HCV-infected patients already appears at the stage of PNALT (persistently normal serum alanine aminotransferase) and is associated with the severity of the clinical stages (i.e., PNALT, chronic hepatitis, liver cirrhosis, and HCC) [65]. In a study investigating the effects of antiviral drugs on the gut microbiota of HCV patients, Ponziani et al. reported significant improvement in gut microbiota dysbiosis after successful DAA treatment of HCV-infected patients [68]. In our preliminarily study, all four patients had a reduction in periodontal bacteria after SVR. Whether HCV elimination affects both gut bacteria and oral periodontal bacteria is a question that needs further investigation.

Among the subjects of this study, case No. 4 (77 years old, male), a cirrhotic patient with HCV infection who had been treated for HCC (see Table 1), was recommended by an oral surgeon to a hepatologist for DAA treatment. He had never received any explanation from his family doctor about antiviral treatment with DAA therapy. So, the oral surgeon explained to him that DAA treatment is the standard treatment for hepatitis C in Japan and that HCV elimination reduces the recurrence of HCC and may also cure OLP and then referred him to a hepatologist. The patient was treated with DAA which resulted in SVR and disappearance of the OLP lesions. It has been four and a half years since SVR, and the patient's HCC has not recurred.

In recent years, the number of HCV-infected people has been decreasing due to the development of DAA treatment, but the existence of HCV-infected individuals who have not yet been diagnosed and HCV patients who have not received appropriate treatment remains a challenge. It is estimated that about 3 million individuals in Japan are infected with HBV and HCV and about 800,000 are unaware of their infection [5]. Of the HCV-infected individuals in Japan, it is estimated that about 470,000 visit medical institutions, about 300,000 are unaware that they are infected, and about 250,000–750,000 are aware that they are infected with HCV but do not receive treatment [69]. We reported that it is possible for dentists to pick out HCV-infected patients from among those who visit dental clinics for the treatment of dental and oral diseases [17, 18, 70, 71]. The detection of potential HCV-infected patients by dentists as gatekeepers can contribute to the reduction of liver carcinogenesis [72].

The present study has some limitations. First, the sample was very small. Second, the study was not conducted in a control group setting. Future studies need to be large case-control studies to clarify the effects of periodontal bacteria.

## 5. Conclusions

In this pilot study, HCV elimination was found to not only improve OLP but also reduce the number of periodontal bacteria. It is important to emphasize that further studies are needed due to the small number of cases. HCV elimination will bring a new perspective on the importance of oral management since HCV infection may be a risk for worsening periodontal disease.

## Figures and Tables

**Table 1:** Characteristics of the subjects (*n* = 4).

No.	1	2	3	4
Sex	Female	Male	Female	Male
Age	76	72	65	77
Liver diseases	CH-C, after HCC	CH-C, after HCC	CH-C	LC-C, after HCC
HCV genotype/level of HCV RNA	1b/high	1b/high	2a/low	1b/high
Past history of IFN therapy (yes/no)	Yes	No	Yes	No
DAA type	DCV/ASV	DCV/ASV	SOF/RBV	DCV/ASV
HBsAg	Negative	Negative	Negative	Negative
Types of OLP before DAA treatment	Erosive	Erosive	Reticular	Erosive
Sites of OLP before DAA treatment	Bilateral buccal mucosa	Bilateral buccal mucosa and lower lip	Bilateral buccal mucosa, tongue, and sublingual mucosa	Lower lip
Systemic disease other than liver disease	OLP, hypertension, and glaucoma	OLP and diabetes mellitus	OLP	OLP, diabetes mellitus, hypertension, and renal failure
Outcome of OLP after SVR24	Disappearance	Disappearance	Improvement	Disappearance
Smoking history (yes/no)	No	Yes	No	Yes
Alcohol intake (yes/no)	No	Yes	No	No
BMI (kg/m^2^) before DAA treatment	22.7	24.9	25.1	20.1
A habit of brushing teeth after meals (more than twice) (yes/no)	Yes	Yes	Yes	Yes

Patient no. 4 was undergoing dialysis treatment. HCV, hepatitis C virus; IFN, interferon; CH-C, chronic hepatitis C; LC, liver cirrhosis; HCC, hepatocellular carcinoma; DAA, direct-acting antiviral agent; DCV, daclatasvir; ASV, asunaprevir; SOF, sofosbuvir; RBV, ribavirin; HBsAg, hepatitis B surface antigen; OLP, oral lichen planus; SVR, sustained virological response; and BMI, body mass index.

**Table 2:** Comparison of the biochemical and periodontitis bacteria data at baseline and SVR24.

Category [normal range]	Baseline	SVR24
BMI (kg/m^2^) (mean ± SD) [18.5–25.0]	23.2 ± 2.4	22.9 ± 2.5
Obesity (BMI ≧ 25) (*n*, %)	1 (25%)	1 (25%)
AST (U/L) (mean ± SD) [13–30]	43.5 ± 19.1	24.0 ± 11.8
ALT (U/L) (mean ± SD) [7–30]	39.8 ± 20.9	19.0 ± 11.2
LDH (U/L) (mean ± SD) [118–229]	234.0 ± 89.7	218.7 ± 37.5
ALP (U/L) (mean ± SD) [106–322]	385.0 ± 272.3	241.5 ± 112.6
T. pro (g/dL) (mean ± SD) [6.60–8.10]	7.57 ± 0.68	7.27 ± 0.59
Alb (g/dL) (mean ± SD) [4.10–5.10]	3.91 ± 0.38	4.01 ± 0.40
T. Bil (mg/dL) (mean ± SD) [0.40–1.20]	0.74 ± 0.33	0.56 ± 0.10
Fasting glucose (mg/dL) (mean ± SD) [73–109]	90.0 ± 12.2	100.3 ± 20.6
HbA1c (%) (mean ± SD) [4.9–6.0]	5.70 ± 0.26	5.80 ± 0.44
T.cho (mg/dL) (mean ± SD) [142–219]	153.3 ± 20.7	211.7 ± 38.7
RBC (10^4^/*µ*L) (mean ± SD) (Male:435–555 and female: 386–492)	400.0 ± 63.0	446.8 ± 46.5
Hb (g/dL) (mean ± SD) (male: 13.7–16.8 and female: 11.6–14.8)	12.7 ± 2.8	10.4 ± 3.2
WBC (10^2^/*µ*L) (mean ± SD) [33–86]	41.4 ± 5.6	58.1 ± 6.5
Plt (10^4^/*µ*L) (mean ± SD) [15.8–34.8]	12.0 ± 6.9	10.4 ± 3.2
AFP (ng/mL) (mean ± SD) [0.0–7.0]	64.2 ± 122.3	4.8 ± 4.7
Total number of bacteria in saliva (copies/10 µl) (mean ± SD)	11.622,500 ± 7.786,957	5.975,000 ± 3.765,966
Number of *A. actinomycetemcomitans* in saliva (copies/10 µl) (mean ± SD)	10 ± 0	10 ± 0
Number of *P. intermedia* in saliva (copies/10 µl) (mean ± SD)	8.596 ± 11.263	9.513 ± 12.872
Number of *P. gingivalis* in saliva (copies/10 µl) (mean ± SD)	2.440 ± 2.791	1.445 ± 2.705
Number of *T. forsythia* in saliva (copies/10 µl) (mean ± SD)	7.570 ± 9.176	1.530 ± 2.023
Number of *T. denticola* in saliva (copies/10 µl) (mean ± SD)	1.470 ± 1.580	770 ± 1.355
Number of *F. nucleatum* in saliva (copies/10 µl) (mean ± SD)	591.275 ± 758.110	191.750 ± 326.387
Risk of *A. actinomycetemcomitans* (ratio of the total bacterial count > 0.006%) (yes, %)	0 (0%)	0 (0%)
Risk of *P. intermedia* (ratio of the total bacterial count > 0.1%) (yes, %)	1 (25%)	2 (50%)
Risk of *P. gingivalis* (ratio of the total bacterial count > 0.1%) (yes, %)	0 (0%)	0 (0%)
Risk of *T. forsythia* (ratio of the total bacterial count > 0.1%) (yes, %)	2 (50%)	0 (0%)
Risk of *T. denticola* (ratio of the total bacterial count > 0.1%) (*n*, %)	1 (25%)	0 (0%)
Risk of *F. nucleatum* (ratio of the total bacterial count > 5%) (yes, %)	1 (25%)	1 (25%)
Risk for at least one of the 3 types of red complex bacteria (yes, %)	2 (50%)	0 (0%)
*P. gingivalis* fimA		
・Genotype II (*n*, %)	1 (25%)	1 (25%)
・Genotype III (*n*, %)	1 (25%)	1 (25%)
・Below detection sensitivity limits (*n*, %)	2 (50%)	2 (50%)

SD, standard deviation; SVR, sustained virological response; BMI, body mass index; AST, aspartate aminotransferase; ALT, alanine aminotransferase; LDH, lactate dehydrogenase; ALP, alkaline phosphatase; T. pro, total protein; Alb, albumin; T. Bil, total bilirubin; T. cho, total cholesterol; RBC, red blood cell; Hb, hemoglobin; WBC, white blood cell; Plt, platelet; and AFP, alpha-fetoprotein.

## Data Availability

All the datasets generated and analyzed in the present study are included in this published article.

## Abbreviations

HCV: Hepatitis C virus
HBV: Hepatitis B virus
HCC: Hepatocellular carcinoma
OLP: Oral lichen planus
IFN: Interferon
DAA: Direct-acting antiviral agent
SVR: Sustained virological response
BMI: Body mass index
RBC: Red blood cell
WBC: White blood cell
Plt: Platelet
Hb: Hemoglobin
ALT: Serum alanine aminotransferase
AST: Aspartate aminotransferase
LDH: Lactate dehydrogenase
ALP: Alkaline phosphatase
T. Bil: Total bilirubin
T. cho: Total cholesterol
T. pro: Total protein
Alb: Albumin
AFP: Alpha-fetoprotein.
